# Complete genomes of common bacterial hosts of RNA bacteriophage Φ6

**DOI:** 10.1128/mra.01000-24

**Published:** 2024-12-10

**Authors:** Taylor Andrews, Siobain Duffy

**Affiliations:** 1 Microbial Biology Graduate Program, Rutgers University, New Brunswick, New Jersey, USA; 2 Department of Ecology, Evolution, and Natural Resources, School of Environmental and Biological Sciences, Rutgers University, New Brunswick, New Jersey, USA; University of Maryland School of Medicine, Baltimore, Maryland, USA

**Keywords:** *Pseudomonas*, phi6, cystovirus, experimental evolution

## Abstract

Bacterial plant pathogens *Pseudomonas savastanoi* pv. *phaseolicola*, *P. syringae* pv. *tomato*, *P. syringae* pv. *atrofaciens*, and *P. oleovorans* East River isolate A serve as common laboratory hosts for bacteriophage Φ6, a widely used model organism for enveloped viruses. Here, we report complete genome sequences for strains of these bacterial hosts.

## ANNOUNCEMENT

Bacteriophage Φ6 and its bacterial hosts comprise a widely used model system (1) and Φ6 serves as an excellent surrogate for eukaryotic, enveloped viruses including SARS-CoV-2 (2). We report the complete sequences of Φ6 bacterial hosts *Pseudomonas savastanoi* pv. *phaseolicola*, *P. syringae* pv. *tomato*, *P. syringae* pv. *atrofaciens*, and *P. oleovorans* East River isolate A and their plasmids (3). These genomes were determined through hybrid *de novo* assembly, combining Illumina and Nanopore reads.

Bacterial strains were acquired from Paul Turner (Yale), who obtained them from Gregory Martin (*P. syringae* pv. *tomato* [GM 113], *P. syringae* pv. *atrofaciens* [GM 2231]), Leonard Mindich (*P. oleovorans* East River isolate A) (3), and the American Type Culture Collection (*P. savastanoi* pv. *phaseolicola* HB10Y, ATCC no.21781).

Glycerol stocks (40%) were stored at −70°C. Bacterial cells from freezer stocks were streaked onto LC agar plates and incubated at 25°C for 2 days, and single colonies were used to inoculate 10  mL of LC broth and grown overnight at 25°C, 110  rpm. Cell pellets from overnight cultures were sent for sequencing by SeqCenter (Pittsburgh, PA) and Plasmidsaurus (Eugene, OR).

The Illumina Whole Genome Sequencing pipeline was used at SeqCenter. DNA was extracted using ZymoBIOMICS DNA extraction kits. Sequencing libraries were prepared with Illumina DNA Prep kits and custom IDT 10 bp unique dual indices with a 280 bp target insert size. Paired-end reads (2 × 151 bp) were produced using an Illumina NovaSeq X Plus sequencer. Bcl-convert (v4.2.4, Illumina) performed demultiplexing, quality control, and adapter trimming.

Cell pellets were washed with 1X PBS, resuspended in Zymo 1X DNA/RNA Shield, and shipped to Plasmidsaurus for Nanopore sequencing. DNA was isolated using Zymo *Quick*-DNA Fungal/Bacterial Miniprep Kits.

An amplification-free long-read sequencing library was prepared using v14 library prep chemistry (Rapid Barcoding Kit 96 V14). Library selection included 0.6 × bead cleanup to remove highly degraded fragments <450 bp. The base calling algorithm used was ont-dorado-for-promethion (v7.1.4, Oxford Nanopore Technologies) on superaccurate mode and read quality control used a minimum Q-score of 10 with adapters trimmed by MinKNOW (v23.07.12, Oxford Nanopore Technologies). Sequencing was performed using PromethION P24 with an R10.4.1 flow cell.

Sequencing and assembly quality and statistics are described in Table 1. Hybrid assembly of Illumina and Nanopore reads was performed using Unicycler (Galaxy Version 0.5.0+galaxy2) (4). Unicycler trims overlaps and rotates replicons during polishing; no manual trimming or rotation was performed. Contigs were polished using Galaxy Workflow: Assembly polishing-upgraded (version:1)) (5) to yield complete genome assemblies. Chromosome and plasmid sequences were annotated using NCBI PGAP (v6.8) (6). Default parameters were used for all software except where otherwise noted. We realigned Illumina reads to these polished genomes (BWA-MEM Galaxy 2.2.1+galaxy1) (7) (visualized with IGVtools v2.16.1 (8)), with the site of linearization offset by ~20,000 bp to see if the linearization site missed bases in the true, circular molecule. When insertions were found, bases were added to the end of the polished sequence (GenBank v1 is prior to base addition; v2 includes added bases). Table 1 details the final, circular assemblies.

**TABLE 1:** Nanopore and Illumina sequencing data and assembly statistics

Sample	Nanopore sequencing				Illumina sequencing			Contig	GenBank accession #	Total size (bp)	GC content	# predicted genes
	Read N_50_ (bp)	Total # of reads	Raw coverage	SRA accession	Total read pairs	Average coverage	SRA accession					
*Pseudomonas savastanoi* pv. *phaseolicola* strain HB10Y	14674	112612	140×	SRR30258005	5535612	273×	SRR30258009	Chromosome	CP166925.2	5912513	58%	5380
								Plasmid unnamed1	CP166927.2	41148	55%	47
								Plasmid unnamed2	CP166926.2	131970	54%	149
*Pseudomonas syringae* pv. *tomato* strain GM 113	14398	48599	55×	SRR30258004	5447387	258×	SRR30258008	Chromosome	CP166920.2	6146598	59%	5642
								Plasmid unnamed1	CP166922.2	79050	55%	86
								Plasmid unnamed2	CP166921.2	104606	58%	108
*Pseudomonas oleovorans* strain ERA	15698	27734	51×	SRR30258003	5630386	376×	SRR30258007	Chromosome	CP166923.2	4487073	62%	4308
								Plasmid unnamed1	CP166924.2	68455	62%	82
*Pseudomonas syringae* pv. *atrofaciens* strain GM 2231	13445	51877	61×	SRR30258002	6090520	308×	SRR30258006	Chromosome	CP166624.2	5933953	59%	5169

## Data Availability

Results are available as BioProject PRJNA1142166. Accession numbers are included in Table 1
